# Downregulation of Histone H3 Lysine 9 Methyltransferase G9a Induces Centrosome Disruption and Chromosome Instability in Cancer Cells

**DOI:** 10.1371/journal.pone.0002037

**Published:** 2008-04-30

**Authors:** Yutaka Kondo, Lanlan Shen, Saira Ahmed, Yanis Boumber, Yoshitaka Sekido, Bassem R. Haddad, Jean-Pierre J. Issa

**Affiliations:** 1 Division of Molecular Oncology, Aichi Cancer Center Research Institute, Nagoya, Japan; 2 Department of Leukemia, The University of Texas at M.D. Anderson Cancer Center, Houston, Texas, United States of America; 3 Lombardi Comprehensive Cancer Center, Department of Oncology, Georgetown University Medical Center, Washington, D.C., United States of America; 4 Lombardi Comprehensive Cancer Center, Department of Obstetrics and Gynecology, Georgetown University Medical Center, Washington, D.C., United States of America; University of Munich and Center of Integrated Protein Science, Germany

## Abstract

**Background:**

Modifications of the histone amino-terminal tails affect access of regulatory factors and complexes to chromatin and thereby influence biological processes. Cancer cells are characterized by prominent epigenetic dysregulation, including histone modifications. However, the functional roles of the histone methyltransferases (HMT) in cancer remain unclear.

**Methodology/Principal Findings:**

We studied RNAi-based inhibition (knockdown, KD) of 2 different H3K9 HMTs, SUV39H1 and G9a. Knockdown of the 2 HMTs in PC3 cancer cell line markedly inhibited cell growth and caused profound morphological changes with loss of telomerase activity and shortened telomeres. SUV39H1 KD cells showed substantial increase in G2/M fraction. G9a KD cells showed increased DNA content (1.7-fold in 2 independent clones) compared with FACS analyses to control. Karyotype analyses showed that this was due to an increased number of chromosomes (from 61 to 102) in G9a KD cells compared to parental PC3. Intriguingly, we found abnormal centrosome morphology and number in about 25% of the G9a KD cells, while centrosomes were morphologically normal in control cells. Microarray analyses after KD of SUV39H1 or G9a showed very few genes up-regulated among the 39,000 genes. The silenced tumor-suppressor genes *p16* and *RASSF1A* were not activated in KD cells.

**Conclusions/Significance:**

These data suggest that the 2 HMTs, SUV39H1 and G9a are required to perpetuate the malignant phenotype. Furthermore, G9a plays a critical role in regulating centrosome duplication presumably through chromatin structure rather than through affecting gene expression in cancer cells. Targeting these histone methyltransferases may be of therapeutic benefit in cancers.

## Introduction

Chromatin assembly is a critical process related to DNA replication, gene expression and progression through the cell cycle. Specific modifications are associated with certain DNA template-mediated processes [1]. Methylation of histone H3 lysine 9 (H3K9) is one of the most well-studied histone modifications. After the initial identification of SUV39h1 in addition to the highly related SUV39h2 as a H3K9-specific histone methyltransferase (HMT) [2], at least three other HMTs, G9a, ESET/SETDB1 and EuHMTase1, have been recognized as HMTs for H3K9 in mammals [3]–[5]. These enzymes have different affinities for the un-, mono- or dimethylated states and produce different methylation states. Studies in knockout mice for Suv39h1, 2 and G9a revealed that G9a is mainly responsible for monomethylation (1Me) and dimethylation (2Me) of H3K9, whereas Suv39h1 and Suv39h2 direct trimethylation (3Me) of H3K9. Furthermore, 1Me and 2Me on H3K9 primarily reside in euchromatin, while 2Me and 3Me on H3K9 are found within different types of heterochromatin, facultative and constitutive heterochromatin, respectively. These results suggest that 1Me, 2Me, or 3Me at K9H3 occupy distinct chromosome domains and each of the three states of H3K9 methylation plays a unique role in the structural and functional organization of chromosomes.

Regulation of higher-order chromosome structure affects mitotic fidelity and ensures balanced chromosome segregation. Heterochromatin assembly at centromeres facilitates both kinetochore formation and sister chromatid cohesion [6]. Furthermore, the formation of chromatin structures at telomeres also serves to maintain the length of telomere repeats [7]. Studies in fission yeast showed that interaction between 3MeH3K9 and Swi6/HP1 is required for chromosome segregation in mitosis [8]. In mammals, mitotic chromosomes display enriched 2MeH3K9 at centromeric regions and pronounced 3MeH3K9 at pericentric heterochromatin. Knockout of Suv39h1 and Suv39h2 in mouse results in widespread genomic instability and increased incidence of lymphomas, suggesting that 3MeH3K9 is a critical modification to maintain chromosomal environments [2]. 2MeH3K9 has also been implicated in DNA-methylation associated gene silencing [9]–[11], but the enzyme control of this event has not been defined.

Cancer cells are characterized by prominent epigenetic dysregulation, including altered chromatin modification. Previously we found increased level of G9a in human cancers [12], although the functional role of the HMTs overexpression in cancer remains unclear. Here, we show that knockdown (KD) of G9a and SUV39H1 in cancer cells remarkably inhibited cell growth and led to morphologically senescent cells with telomere abnormalities. We found that G9a KD but not SUV39H1 KD induces extensive chromosome instability and centrosome disruption. These data suggest that G9a as well as SUV39H1 are required to maintain the malignant phenotype and could be valid therapeutic targets in human neoplasia.

## Materials and Methods

### Cell Lines, Culture Conditions and Drug Treatment

The prostate cancer cell line PC3, the lung cancer cell line H1299 and the breast cancer cell line MCF7 were obtained from the American Type Culture Collection (Manassas, VA). They were grown in RPMI medium (Invitrogen, Carlsbad CA), plus 10% FBS in plastic tissue culture plates in a humidified atmosphere containing 5% CO_2_ at 37°C. Cells were split 12–24 h before 5-Aza-2′-deoxycytidine (DAC, Sigma, St. Louis, MO) treatment. Cells were then treated with either 1 µM of DAC, or PBS (control) daily for 3 days.

### RNA Interference

We designed a retrovirus vector (RNAi-Ready pSIREN-RetroQ Vector, BD Biosciences) encoding a small hairpin RNA (shRNA) directed against G9a and SUV39H1 in PC3 (target sequences of 5′-AAGATTGAGCCTCCGCTGATT-3′ and 5′-AATCTCAAGTGTGTGCGTATC-3′ for G9a and SUV39H1, respectively). As a control, we used shRNAs for Luciferase (Luc) synthesized by BD Biosciences or shRNA vector without hairpin oligonucleotides.

### Chromatin Immunoprecipitation (ChIP)

ChIP assays were performed based on a modification of previously published methods [13]. Briefly, cells are treated with 1% formaldehyde for 8 min to crosslink histones to DNA. Cell pellets are resuspended in lysis buffer and sonicated 8 sec seven times. The lysate is incubated with 10 µl of anti-K4 dimethylated histone H3, anti-K9 acetylated histone H3, anti-K9 dimethylated histone H3 (Upstate Biotechnology, Charlottesville, VA) and anti-histone H3 antibody (as an internal control, Abcam, Cambridge, UK) at 4°C overnight. Two % of total lysate is used for input control. DNA is extracted by the phenol/chloroform method, ethanol-precipitated, and resuspended in water. ChIP products were used for quantitative PCR with the oligonucleotide primers described in Table S1. For quantitation, TaqMan Q-PCRs were performed in an ABI Prism 7000 instrument (Applied Biosystems, Foster City, CA) in duplicate for the target genes.

### Immunoblotting

Immunoblotting was performed with primary antibodies for anti-G9a (Abcam), anti-SUV39H1, anti-K9 dimethylated histone H3, anti-K9 trimethylated histone H3 (Upstate Biotechnology), histone H3 (Abcam) and ß-actin (Sigma).

### RT-PCR Analyses

Total RNA was isolated using TRIzol (Invitrogen), and 2 µg was reverse transcribed with MLV-reverse transcriptase (Invitrogen). The primer sequences used are described in Table S1. The PCR protocol for RT-PCR was 95°C for 4 min, followed by 35 cycles of 30 sec at 95°C, 30 sec at 55°C and 30 sec at 72°C, and then a 5 min final extension at 72°C. TaqMan Q-PCRs were carried out in duplicate for the target genes G9a, SUV39H1 and GAPDH using probe sets Hs00198710_m1, Hs00162471_m1 and Hs 00266705_gl, respectively (Applied Biosystems).

### Bisulfite Pyrosequencing Methylation Analysis

We performed bisulfite treatment as reported previously [14]. Briefly, 2 µg of genomic DNA were denatured with 2 M NaOH for 10 min, followed by incubation with 3 M sodium bisulfite (pH 5.0) for 16 h at 50°C. After treatment, DNA was purified using a Wizard Miniprep Column (Promega, Madison, WI), precipitated with ethanol, and resuspended in 30 µl of diluted water. We used a highly quantitative method to assess DNA methylation levels based on Pyrosequencing technology [15] (Pyrosequencing AB, Uppsala, Sweden). Primer sequences are described in Table S1.

### SA-ß-gal Analysis

PC3 cells infected with retrovirus encoding G9a-shRNA, SUV39H1-shRNA or control-shRNA were examined for SA-ß-gal activity as previously described [16].

### RNA Microarray Analysis

Total RNA was extracted from cells infected with either G9a-shRNA, SUV39H1-shRNA or control-shRNA as described above. Targets for microarray hybridization were generated from the RNA according to the manufacturer's instructions (Affymetrix, Santa Clara, CA). The human U133A gene chip (Affymetrix) which contains ∼33,000 transcripts was used for gene expression profiling. Hybridization, washing, scanning, and analysis of gene chips were performed according to the manufacturer's instructions. Expression levels were analyzed by the statistical algorithm in the Microarray Analysis Suite (MAS) 5.0 software (Affymetrix) using the default parameters. The data from the control-shRNA treatment and parental PC3 were used as a baseline expression for comparison with either G9a-shRNA or SUV39H1-shRNA treated sample.

### Measurement of Telomerase Activity

Telomerase activity was analyzed by a modified method of the standard telomerase repeat amplification protocol (TRAP), the TRAPeze Telomerase Detection Kit (Millipore, Billerica, MA) according to the manufacturer's instructions. Reaction products were run on a 15% native polyacrylamide gel and stained with ethidium bromide.

### Immunofluorescence Cytogenetic

G9a-shRNA, SUV39H1-shRNA or control-shRNA treated PC3 cells were grown on Falcon culture slides (Becton, Dickinson and Company, Franklin Lakes, NJ). Anti-CENP-A monoclonal antibody (Abcam), anti-K9 dimethylated histone H3 and anti-K9 trimethylated histone H3 polyclonal antibodies (Upstate Biotechnology) were used as primary antibodies. Centrosomes were detected with a γ-tubulin polyclonal antibody (Sigma) using a standard protocol [17]. The primary antibody was detected with Alexa Fluor 488 conjugate goat anti-mouse antibody (Invitrogen), Alexa Fluor 546 conjugate goat anti-rabbit antibody (Invitrogen) or fluorescein-conjugated goat anti-rabbit antibody (Sigma) and the cells were counterstained with 4′,6-diamidino-2-phenylindole (DAPI), and embedded in anti-fade solution (200 mM DABCO, 90% vol/vol glycerol, 20 mM TRIS-HCl, pH8) to reduce photo-bleaching. Scoring of cells and digital image acquisition were performed using a 63× objective mounted on a Leica DMRBE microscope (Leica, Wetzlar, Germany) equipped with optical filters for DAPI and FITC (Chroma Technologies, Brattleboro, VT) and a cooled charge coupled device (CCD) camera (Photometrics, Tucson, AZ). The IPLab software package (Scanalytics Inc, Fairfax, VA) was used for image acquisition and processing. For each of the 3 cell preparations (control, G9a-shRNA or SUV39H1-shRNA treated cells), 100 nuclei were scored by two independent observers.

### Telomere Length Analysis

G9a-shRNA, SUV39H1-shRNA or control-shRNA-treated PC3 cells were evaluated for telomere shortening using the Telo-FISH method to visualize telomeres in interphase cell populations, as described previously [18]. Briefly, FISH analysis was performed using a commercially available Cy3-conjugated peptide nucleic acid (PNA) telomeric probe per the manufacturer's (DAKO Corporation, Carpinteria, CA) instructions. Independent analysis was performed by two blinded observers using a Leica DMRBE microscope equipped with a Cy3 and 4′,6-diamidino-2-phenylindole (DAPI) filters. More than 100 interphase nuclei were visually evaluated, and digital images were obtained using a cooled CCD (charge coupled device) camera.

## Results

**Figure 1:**
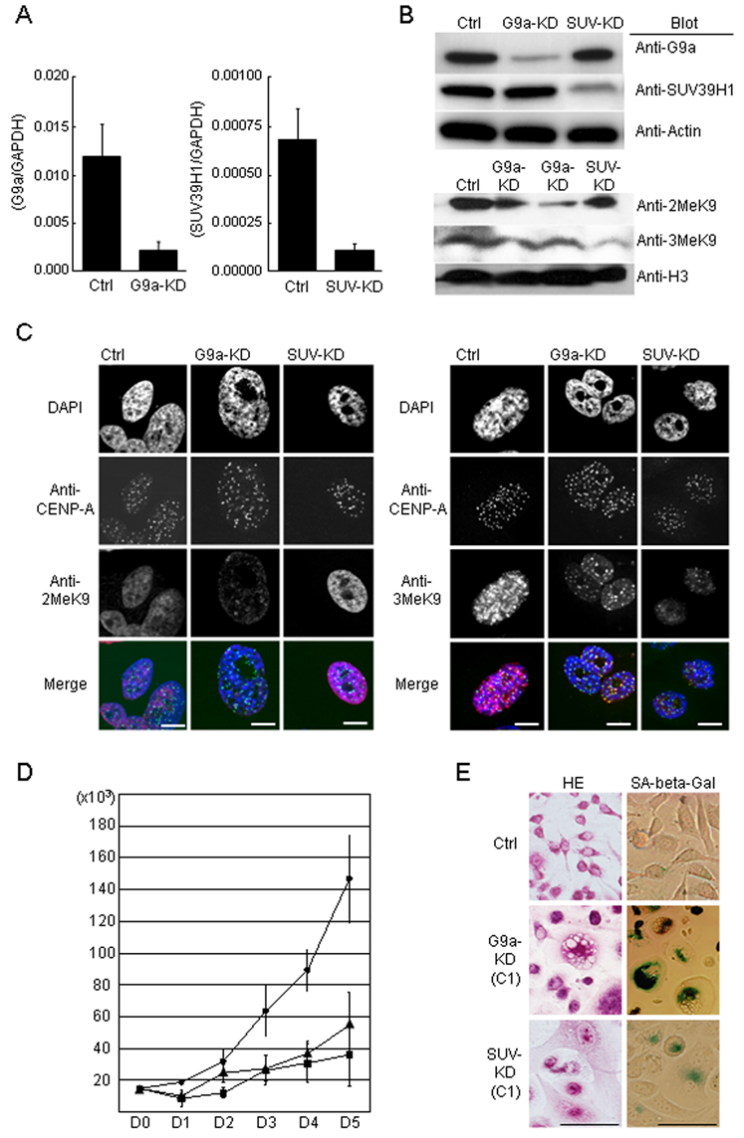
Effects of histone methyltransferases, either G9a or SUV39H1 knockdown (G9a-KD or SUV-KD, respectively). Real-time PCR (A) and Western blotting (B) show that shRNA interference effectively downregulates G9a or SUV39H1 expression. Error bars in real-time PCR indicate standard deviation of the expression in more than 3 independently-isolated clones. Regarding H3 modifications, the G9a-KD (2 independent clones) cells show reduction of overall 2MeH3K9. By contrast, SUV-KD reduced overall 3MeH3K9 but no remarkable change on 2MeH3K9. C, H3-K9 methylation states were analyzed by immunofluorescence with 2MeH3K9 (red, left) or 3MeH3K9 (red, right) specific antibodies in control cells (Ctrl), G9a-KD cells or SUV-KD cells. 2MeH3K9 was broadly detected in control nuclei. The G9a-KD nucleus revealed faint speckles of 2MeH3K9 overlapping with DAPI-dense (blue) loci and the CENP-A staining (green). which represent the pericentric region and centromeric region, respectively. SUV-KD reduced 3MeH3K9 at the heterochromatic foci and did not significantly alter the euchromatic staining of 2MeH3K9 states. Cell size is relative to the 10-µm bar. D, G9a-KD and SUV-KD result in strong growth inhibition. Cells were plated at the same density and counted daily by Trypan blue exclusion. Filled circles, filled triangles and filled squares indicate the growth of the control, the SUV-KD and the G9a-KD cells, respectively. Error bars on the graph indicate the standard deviation from triplicate experiments. E, Downregulation of two histone methyltransferases result in a remarkable morphologic change, which is stained positively for SA-beta-gal, indicating induction of senescence. Cell size is relative to the 50-µm bar.

To evaluate the role of SUV39H1 and G9a in cancers, first we established clones of PC3, in which either of these 2 HMTs were stably downregulated by shRNA. We used PC3, since endogenous SUV39H1 and G9a were highly expressed in this cell. In these knockdown (KD) clones, expression of the 2 HMTs was reduced by 80–90% (Figs. 1A and 1B). KD cells for G9a (G9a-KD) showed reduction of overall 2MeH3K9 and 3MeH3K9. KD cells for SUV39H1 (SUV-KD) reduced overall 3MeH3K9 but no remarkable change on 2MeH3K9 (Fig. 1B). 2MeH3K9 was broadly detected in nuclei of control cells including eu- and heterochromatic region (Fig. 1C). However, the G9a-KD cell nucleus showed selectively impaired broad euchromatic staining of 2MeH3K9 and contained only blurred speckles, which were overlapping with the pericentric region (densely stained with DAPI) and centromeric region (stained with CENP-A). 3MeH3K9 was detected on the pericentric region and centromeric region in control cells. SUV-KD cells showed diminished 3MeH3K9, but not completely, at the heterochromatic foci and did not significantly alter the 2MeH3K9 states (Fig. 1C). G9a-KD did not significantly affect 3MeH3K9 patterns of the pericentric region and centromeric region. These findings were consistent with the previous G9a- deficient ES cell studies [19], [20]. Interestingly, it appeared that G9a-KD affected H3K9me3 status at euchromatic region.

Both SUV-KD and G9a-KD showed markedly inhibited cell growth and profound morphological changes, enlarged and flattened shapes. (Figs. 1D and 1E). Senescence associated ß- galactosidase analysis (SA-ß-gal analysis) demonstrated that this morphological change was consistent with induction of cellular senescence.

**Figure 2:**
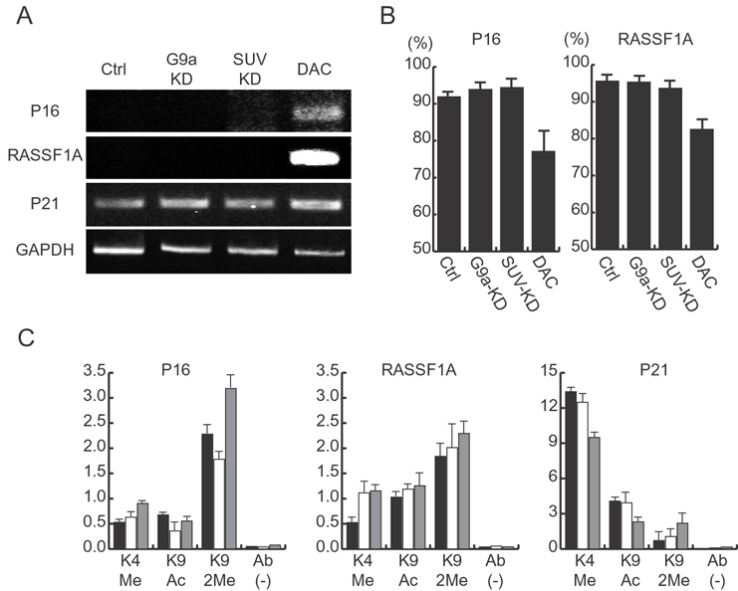
Neither G9a nor SUV39H1 knockdown affects gene expression and epigenetic states of p16 and RASSF1A. A, RNA expression of each gene was examined by RT-PCR. In contrast to 5-Aza-2′-deoxycytidine (DAC) treatment, neither G9a-KD nor SUV-KD reactivates p16 and RASSF1A expression. p21 and GAPDH are used for active gene controls. B, DNA methylation changes on the p16 and the RASSF1A promoters were analyzed by the pyrosequencing quantitative method. Consistent with the expression status for both genes, DNA methylation is decreased by DAC treatment, whereas no changes are observed in either G9a-KD or SUV-KD cells. Error bars on the graph indicate the standard deviation from triplicate experiments. C, Histone modifications studied by ChIP are indicated below the columns. K4Me, K9Ac, K9Me and Ab(-) indicates K4 dimethylation, K9 acetylation, K9 dimethylation and no antibody control, respectively. The y axis represents the ratio of each IP to a core histone H3 immunoprecipitation. Black bars, white bars and gray bars indicate the ChIP from the mock control, the G9a-KD and the SUV-KD cells, respectively. p21 was used as an active gene control, in which active histone marks, K4Me and K9Ac, are high. Error bars on the graph indicate the standard deviation from triplicate experiments.

Induction of cellular senescence in response to stimuli depends primarily on the p53 or p16-pRB pathways [21]. Since p16 is silenced by DNA methylation in PC3 and its p53 status is null, we next examined p16 reactivation in the G9a-KD and SUV-KD cells. By contrast to treatment with the DNA methyltransferase inhibitor 5-aza-2′-deoxycytidine (DAC), we found p16 activation in neither the G9a-KD nor the SUV-KD cells, consistent with no effect on epigenetic status of the promoter region (Fig. 2). We also examined another tumor suppressor gene, *RASSF1A*, which is a target of DNA methylation in PC3, and found that there was no effect on gene reactivation in either the G9a-KD or the SUV-KD cells. Given that DNA methylation, a crucial epigenetic mechanism for silencing tumor suppressor genes in human neoplasia, has been mechanistically linked to histone 2MeH3K9 methylation [9]–[11], these data could be explained by compensation by other HMTs.

To examine target genes of H3K9 methylation dependent silencing by either G9a or SUV39H1, we analyzed genes up-regulated after inhibition of G9a or SUV39H1 using oligonucleotide microarrays. In analyzing the array data, first we selected genes with signal intensities of 50 or less in either parental or control shRNA-treated PC3 (i.e. silenced genes). Then we searched for genes with signal intensities increased more than 2 times after KD of either G9a or SUV39H1. After removal of self-inconsistent probes among the same gene, we could identify only 4 and 6 genes (3 genes are common) in the G9a-KD and the SUV-KD cells, respectively. We examined those 7 genes by RT-PCR and found that only 2 genes, *Klotho* and *growth differentiation factor 8* (*GDF8*), were truly silenced in the control cells and up-regulated in both G9a-KD and SUV-KD cells (data not shown). Thus, very few genes are up-regulated after inhibition of the 2 HMTs in PC3. Although, G9a and SUV39H1 are generally involved in gene silencing, we found that 12 and 2 genes (2 genes were common) were down-regulated in the G9a-KD and the SUV-KD cells, respectively (Table S2). Down-regulation of those few genes might be due to the secondary effects of G9a-KD and the SUV-KD.

**Figure 3:**
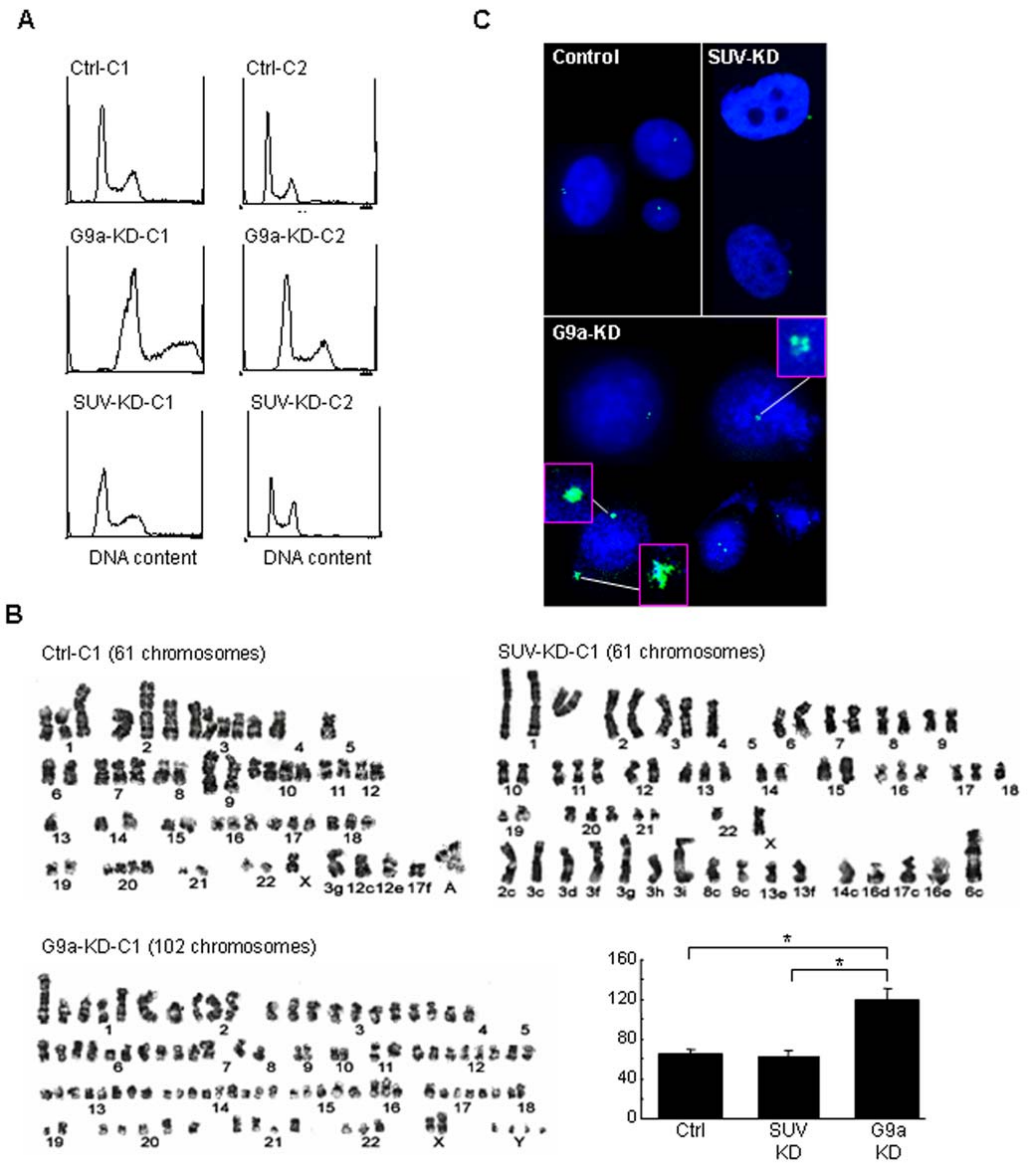
Downregulation of G9a methyltransferase induces genomic instability. A, FACS analyses showing DNA content is increased 1.5–2 times in the G9a-KD cells compared to the mock control in two independently-isolated clones (G9a-C1 and -C2). The SUV-KD cells show a high amount of G2M cells compared to the mock control. B, karyotype analyses showed 61, 102 and 61 chromosomes in the control PC3, the G9a-KD and the SUV-KD cell lines, respectively. Chromosome number is remarkably increased in the G9a-KD cells. Numbers of the chromosome were counted in more than 8 nucleus of each KD cell. G9a-KD cells showed significantly increased chromosome numbers than the SUV-KD cells and control cells. Y axis indicates number of chromosome. Error bars indicate standard deviation. *, *P*<0.01. C, Nuclei were stained with DAPI. Centrosomes were detected using an antibody against γ-tubulin. In the control cells and SUV-KD cells, 1–2 centrosomes appearing as point-like structures are seen in each cell. In the G9a-KD cells, abnormal centrosome morphology and number, suggestive of centrosome amplification was observed in about 25% of the cells. Abnormally amplified centrosomes are magnified in the boxes.

Despite minimal effects on gene expression, markedly inhibited cell growth was observed in both the G9a-KD and the SUV-KD cells. We examined these cells by FACS analyses to determine the mechanism involved. We found that the G9a-KD cells showed increased DNA content (1.7-fold in 2 independent clones) and the SUV-KD cells evidenced substantial increases in G2/M fraction (from 29% to 40%) compared to the control (Fig. 3A). Consistent with these data, karyotype analyses (Fig. 3B) revealed an increased number of chromosomes in the G9a-KD cells compared to the SUV-KD cells and the parental PC3 cell line (Fig. 3B). Numbers of the chromosome were counted in more than 8 nucleus of each KD cell (Fig. 3B). G9a-KD cells showed significantly increased chromosome numbers (average, 119 chromosomes, *P*<0.01) than the SUV-KD cells (63 chromosomes) and control cells (65 chromosomes). This was true regardless of the cell line types used. Both stable G9a-KD clones of the breast cancer cell line MCF7 and the lung cancer cell line H1299 also showed an increased number of chromosomes compared to control cells (MCF7-control vs MCF7-G9a-KD, 60 chromosomes vs 98 chromosomes; H1299-control vs H1299-G9a-KD, 72 chromosomes vs 104 chromosomes; Figure S1).

Centrosomes are essential for proper cellular polarity and balanced distribution of chromosomes [22]. We used an antibody against γ-tubulin to evaluate the centrosomes in the KD cells. Although the majority of the cells in both control and G9a-KD showed normal (1 to 2) centrosome numbers, we detected centrosome amplification in about 25% of the cells in the G9a-KD cells. This was not detected in the control cells and SUV-KD cells (Fig. 3C). Centrosome abnormality was also observed in both MCF7-G9a-KD cells and H1299-G9a-KD cells in around 20% of the cells (Figure S1). Thus, G9a might play a critical role in regulating centrosome duplication, presumably through chromatin structure rather than by affecting gene expression in cancer cells.

**Figure 4:**
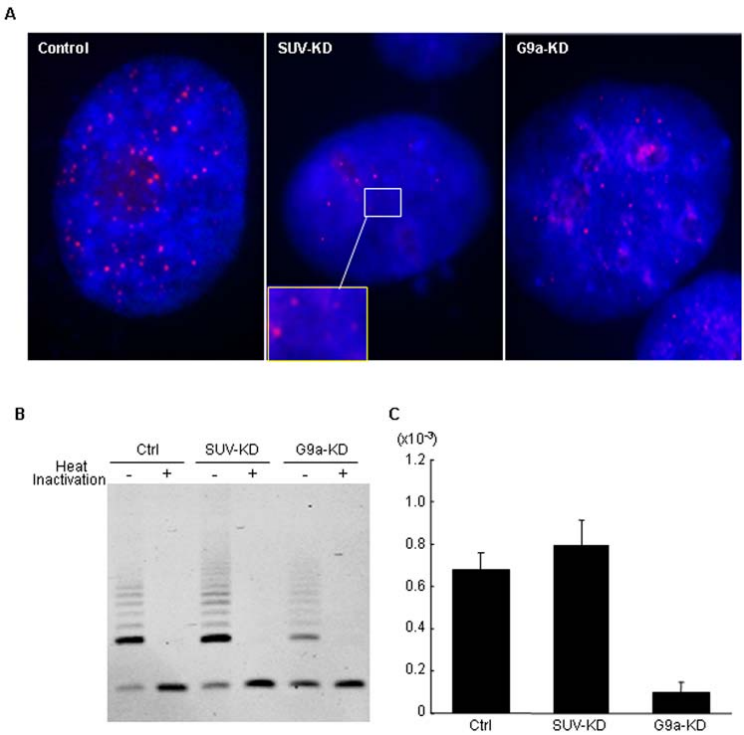
Disruptions of telomere function in the G9a-KD and the SUV-KD cells. A, Interphase nuclei were hybridized with Cy3-conjugated peptide nucleic acid (PNA) telomeric probe and counterstained with DAPI. Marked reduction in the telomere signal is observed in interphase nuclei from KD cells as compared to the nuclei from the control line. Shown in the insets are the enlarged images of the indicated areas. B, Telomerase activity is assessed by TRAP assay. Samples with telomerase activity produce a 6-base incremental ladder of TRAP products on the gels. Internal control bands appear at the bottom of the gel. A reduced signal intensity of telomerase activity is observed in the G9a-KD cell lines. The signal is not observed when the extracts are pretreated with heat to abolish the protein components of telomerase, suggesting the telomerase specificity of the signal measured by this assay. C, Expression level of hTERT was measured by real-time PCR. hTERT expression is decreased in the G9a-KD cells compared to those in the control PC3 and the SUV-KD cells. Error bars indicate standard deviation of the 3 independent assays.

The chromatin structure at telomeres is also important to maintain the length of telomere repeats [7]. We investigated telomere function in the G9a-KD and the SUV-KD cells. First, we used telo-FISH assay to evaluate telomere length. Significant reduction in the telomere signal intensity was observed in the G9a-KD and SUV-KD treated PC3 cells, as compared to the control cells suggesting that regulation of telomere length was disrupted in these KD cells. A representative example of the decrease in the telomere signal is shown in figure 4A. We next quantified telomerase activity in those cells by the TRAP assay and found reduction of telomerase activity in the G9a-KD cell line (Fig. 4B). Therefore, the shortened telomere length in the G9a-KD cells might be the result of altered telomerase activity. The decreased level of hTERT expression in the G9a-KD cells lends support to these findings (Fig. 4C). In contrast, the SUV-KD cells showed similar levels of telomerase activity to the control cells, indicating that telomere dysfunction in the SUV-KD cells might depend on mechanisms other than hTERT expression, probably due to alterations of H3K9 methylation status at telomeric heterochromatin.

## Dsicussion

The current study demonstrates that expression of SUV39H1 and G9a is important to support the growth of malignant cells, although they play distinct roles in cancer cells. Unexpectedly, very few genes are up-regulated after inhibition of the 2 HMTs, suggesting that H3K9 methylation associated silencing, which has been mechanistically linked to DNA methylation [9]–[11], is stable once it is established in cancer cells, possibly through the action of multiple HMTs.

Centromere mediates multiple segregation functions, including kinetochore formation, spindle-mediated movements, sister cohesion and a mitotic checkpoint [6]. The pericentric heterochromatin architecture plays crucial roles in chromosomal segregation as well as in establishing transcriptional repression. The loss of Suv39h1 and Suv39h2 HMTases in mouse model abolished H3-K9 methylation at pericentric heterochromatin and induced chromosomal instability. However, single gene disruptions for either Suv39h1 or Suv39h2 did not appear to affect viability and fertility of mutant mice, suggesting these two enzymes are redundant [2]. Study in G9a knockout mouse showed that G9a was necessary for embryonic development or differentiation and embryonic growth defect in G9a-deficient ES cells might be due to apoptotic cell death but not cell cycle arrest. In that model, chromosomal instability was not perceived in the knockout cells [19]. We found here that in cancer cells, which often harbor aberrant numbers of chromosome, G9a KD induced centrosome disruption and more extensive chromosome instability, which resulted in inhibition of cell growth and cellular senescence. It appeared that 3MeH3K9 was also diminished at euchromatic region in the G9a KD cells. This might be due to the drastic decreasing of 2MeH3K9, which is prerequisite for modification of tri-methylation on the loci. These data suggested that the role of G9a in cancer cells is critical and appears to be protecting further chromosomal disruption. By contrast, single Suv39H1 KD partially abolished 3MeH3K9 at pericentric region but could not induce chromosomal instability, probably due to redundant roles for SUV39H HMTases [2].

Recent reports demonstrated that centromeric chromatin specific combinations of histone modifications and the three-dimensional organization of chromosomes could also be important for recruitment of cohesion complexes to heterochromatin near sister kinetochores [23] [24]. 2MeH3K9 chromatin, which is present in the inner kinetochore space between mitotic sister chromatids and in regions that flank centromeric chromatin, could be attributable to the position of CENP-A toward the poleward face of the mitotic chromosome [25]. The decreased level of 2MeH3K9 on the flanking region of centromere chromatin by G9a KD might affect the three-dimensional organization of centromere chromosomes, resulting in the chromosomal instability we found here.

Centrosomes start to duplicate at the late G1/early S phase of the cell cycle, and two functional centrosomes are formed during G2 [22]. If cells fail to undergo cytokinesis after DNA synthesis and the next cell cycle resumes, they might have twice the normal DNA content and centrosome number. Thus, the current data suggested that failure in cytokinesis might be an explanation for the abnormalities in chromosomal number and centrosomes in the G9a-KD cells.

G9a appears to be required for hTERT expression and telomere maintenance. SUV39H1 is also required for control telomere regulation. In mouse model, embryonic fibroblast from mice null with both Suv39h1 and Suv39h2 showed abnormal telomere elongation [26]. Abrogation of the two HMTs resulted in loss of heterochromatic features at telomeres in embryonic stem cells and mouse embryonic fibroblasts. Our data suggested that SUV39H1 KD in cancer cells have shorter telomeres. The reasons why we found the shortening of the telomeres in SUV39KD cells by contrast to the previously results might be due to the cell types studied (i.e. normal cells versus cancer cells), since telomere function was generally aberrantly regulated in cancer cells [27]. Further studies need to address the mechanistic links between G9a, SUV39H1 and chromosomal/telomere structures as well as hTERT expression in cancer cells. It also might be interesting to see whether the individual effects of G9a and SUV39H1 are cooperative when they are both depleted, and whether reintroduction of the two HMTs reverts the phenotype. Nevertheless, targeting these histone methyltransferases could be of therapeutic benefit in cancer treatments.

## Supporting Information

Figure S1 Downregulation of G9a methyltransferase in MCF7 and H1299 cell lines. A, Real-time PCR shows that shRNA interference effectively downregulates G9a in MCF7 and H1299. Error bars in real-time PCR indicate standard deviation of the expression in 3 independently isolated clones. B, FACS analyses showing DNA content is increased 1.5–2 times in the G9a-KD cells compared to the mock control in an isolated clone. Increased DNA content was observed in two independently-isolated clones (data not shown). C, karyotype analyses showed small and increased chromosomes in both MCF7-G9a-KD and H11299-G9a-KD cell lines. D, Centrosomes were detected using an antibody against γ-tubulin. In the control cells, 1–2 centrosomes appearing as point-like structures are seen. Abnormal centrosome morphology and number are observed in around 20% of the G9a-KD cells.
(2.67 MB EPS)

Table S1 Summary of the primers
(0.04 MB DOC)

Table S2 List of up-regulated and down-regulated genes in G9a-KD or SUV39H1-KD
(0.05 MB DOC)
