# Supplementary material for: Downregulation of Histone H3 Lysine 9 Methyltransferase G9a Induces Centrosome Disruption and Chromosome Instability in Cancer Cells
Source: PLoS One. 2008 Apr 30;3(4):e2037. doi: 10.1371/journal.pone.0002037 (PMC2323574; doi:10.1371/journal.pone.0002037)
Supplement: Table S1 — Summary of the primers (0.04 MB DOC) [file pone.0002037.s002.doc]

Table S1. Summary of the primers

| Genes | Primer sequences | ChipQ-PCR or pyrosequencing probes |
| --- | --- | --- |
| qChIP |  |  |
| P16 | Forward  GGGCGGATTTCTTTTTAACAGA  Reverse  CGCCTGCCAGCAAAGG  60°C | TGAACGCACTCAAAC |
| RASSF1A | Forward  GGACCCTCTTCCTCTAGCACAGT  Reverse  GCACCACGCGGAGATACC  60°C | TGGCCTCCAGAAAC |
| P21 | Forward  GCCAACTCATTCTCCAAGTAAAAAA  Reverse  GTGCGCTGGACACATTTCC  60°C | CCAGATTTGTGGCTCACT |
| RT-PCR |  |  |
| P16 | Forward  CAACGCACCGAATAGTTACGG  Reverse  GCGCAGTTGGGCTCCG  55°C |  |
| RASSF1A | Forward  ACGTGGTGCGACCTCTGTG  Reverse  CGCAACAGTCCAGGCAGAC  55°C |  |
| P21 | Forward  GCAGAGGAAGACCATGTGGAC  Reverse  CGGCGTTTGGAGTGGTAGAA  55°C |  |
| GAPDH | Forward  TCCCATCACCATCTTCCAG  Reverse  ATGAGTCCTTCCACGATACC  55°C |  |
| Pyrosequencing |  |  |
| P16 | Forward  GGTTGTTTTYGGTTGGTGTTTT  Reverse(5’- biotinylated)  ACCCTATCCCTCAAATCCTCTAAAA  58/56/54/52°C | TTTTTGTTTGGAAAGAT |
| RASSF1A | Forward  GGGGGAGTTTGAGTTTATTGA  Reverse(5’- biotinylated)  CTACCCCTTAACTACCCCTTCC  55°C | GGGTAGTATTAGGTTGGAG |

Temperature described below the primer sequences indicates annealing temperature for each reaction.
