# Supplementary material for: Downregulation of Histone H3 Lysine 9 Methyltransferase G9a Induces Centrosome Disruption and Chromosome Instability in Cancer Cells
Source: PLoS One. 2008 Apr 30;3(4):e2037. doi: 10.1371/journal.pone.0002037 (PMC2323574; doi:10.1371/journal.pone.0002037)
Supplement: Table S2 — List of up-regulated and down-regulated genes in G9a-KD or SUV39H1-KD (0.05 MB DOC) [file pone.0002037.s003.doc]

**Table S2.** List of up-regulated and down-regulated genes in G9a-KD or SUV39H1-KD

| Gene Name | Accession No. | Control * | G9a-KD * | SUV-KD * |
| --- | --- | --- | --- | --- |
| Up-regulated Genes in G9a-KD and/or SUV39H1-KD | | | | |
| ABCG1 | NM_004915 | 19.551 | 123.931 | 118.472 |
| SCIN | NM_033128 | 32.2984 | 42.61 | 130.149 |
| ITGA1 | X68742 | 33.7627 | 52.1633 | 112.461 |
| KL | NM_004795 | 36.3001 | 122.249 | 116.087 |
| EHF | NM_012153 | 41.5624 | 95.5524 | 304.859 |
| GHR | NM_000163 | 49.7365 | 62.4218 | 224.976 |
| GDF8 | NM_005259 | 5.77876 | 15.9872 | 11.3961 |
| Down-regulated Genes in G9a-KD and/or SUV39H1-KD | | | | |
| RAB27A | AA743462 | 114.193 | 34.2377 | 81.5586 |
| CXCL5 | NM_002994 | 397.703 | 20.5141 | 15.6848 |
| IKIP | BG498328 | 123.931 | 41.3813 | 75.1224 |
| TFPI | AF021834 | 610.79 | 32.3921 | 561.73 |
| ZFX | NM_003411 | 138.417 | 42.5706 | 123.638 |
| TCF8 | AI806174 | 277.677 | 45.0038 | 229.583 |
| DMXL1 | NM_005509 | 118.765 | 49.0613 | 131.286 |
| FLJ21159 | NM_024826 | 134.256 | 49.8936 | 96.539 |
| MGC34132 | AK023140 | 100.894 | 44.4094 | 56.2216 |
| ELOVL2 | BF508639 | 269.016 | 21.4033 | 29.3516 |
| IRLB | BE268538 | 103.309 | 39.1055 | 82.7849 |
| TMEM5 | BF224146 | 159.008 | 44.815 | 78.1199 |

*, Signal intensities in each manipulated cell on the microarray.
